# Ni-Coated Diamond-like Carbon-Modified TiO_2_ Nanotube Composite Electrode for Electrocatalytic Glucose Oxidation

**DOI:** 10.3390/molecules27185815

**Published:** 2022-09-08

**Authors:** Yi Kang, Xuelei Ren, Yejun Li, Zhiming Yu

**Affiliations:** 1 School of Materials and Engineering, Central South University, Changsha 410083, China; 2 The Third Xiangya Hospital, Central South University, Changsha 410013, China; 3 School of Physics and Electronics, Central South University, Changsha 410083, China

**Keywords:** composite electrode, DLC film, TiO_2_ nanotube, Ni, non-enzymatic glucose sensor

## Abstract

In this paper, a Ni and diamond-like carbon (DLC)-modified TiO_2_ nanotube composite electrode was prepared as a glucose sensor using a combination of an anodizing process, electrodeposition, and magnetron sputtering. The composition and morphology of the electrodes were analyzed by a scanning electron microscope and energy dispersive X-ray detector, and the electrochemical glucose oxidation performance of the electrodes was evaluated by cyclic voltammetry and chronoamperometry. The results show that the Ni-coated DLC-modified TiO_2_ electrode has better electrocatalytic oxidation performance for glucose than pure TiO_2_ and electrodeposited Ni on a TiO_2_ electrode, which can be attributed to the synergistic effect between Ni and carbon. The glucose test results indicate a good linear correlation in a glucose concentration range of 0.99–22.97 mM, with a sensitivity of 1063.78 μA·mM^−1^·cm^−2^ and a detection limit of 0.53 μM. The results suggest that the obtained Ni-DLC/TiO_2_ electrode has great application potential in the field of non-enzymatic glucose sensors.

## 1. Introduction

The accurate detection of glucose is of great importance to blood glucose control, biochemical analysis, and food safety for diabetic patients [1]. The widely used enzymatic glucose detection is limited by the biochemical characteristics of the enzyme and is very sensitive to temperature, pH, humidity, and other conditions. In order to improve the accuracy of enzyme sensor detection, the development of new non-enzymatic glucose sensors has become a research focus in recent years [2,3]. Varied materials have been employed as substrates in glucose sensors. TiO_2_ nanotubes (TNTs), as a new functional material in the field of biosensors, can be prepared at low temperatures and their tube length and diameter are easy to accurately control, which makes them an excellent carrier electrode for nanometer-sensitive materials [4]. Moreover, anodized TNTs are non-toxic with a good biological affinity and large specific surface area. In addition, their directional tubular and ordered hollow structures can provide sufficient adsorption space [5]. However, the low electron conduction efficiency of TiO_2_ nanotubes due to their semiconductor nature significantly restricts their wide application, where additional modifications are generally required to increase the conductivity.

As for the catalytic materials for glucose oxidation, metal nanoparticles have been widely studied due to their high reactivity and simple preparation among non-enzymatic sensors. Among them, the earth-abundant and non-toxic Ni has a low cost and a strong specific catalytic ability to oxidize glucose, particularly in alkaline environments, compared with Pt, Au, and Cu [3,6]. At present, most Ni-based sensors constructed from Ni nanomaterials are mainly used to form a higher valence hydroxide (NiOOH) in alkaline media to carry out the catalytic oxidation process. Ni nanomaterials are usually loaded on graphene, carbon nanotubes, carbon nanofibers, disordered graphite carbon, diamond, and other carbon-based carrier electrodes using chemical or electrochemical deposition [7,8,9,10,11,12]. Compared with the Ni plate, Ni nanoparticle-modified electrodes can significantly improve glucose detection sensitivity. For instance, Zeng et al. [13,14] and Liu et al. [15,16,17] loaded Ni nanoparticles and Ni/Cu nanoparticles on TiO_2_ nanotubes by electrodeposition, respectively, with excellent detection limits and sensitivity. However, when metal nanomaterials are loaded on the electrode by chemical or electrochemical deposition, the chemical bonding between the metal nanoparticles and carbon substrates is generally not so strong, resulting in a high interfacial migration energy barrier of electrons. Moreover, the metal nanoparticles are prone to migrate with aggregation, which greatly affects the activity and stability of the electrode [18]. Diamond-like carbon (DLC) film has excellent biocompatibility and chemical stability. It is a biologically inert material, which has good mechanical and friction resistance properties. Diamond-like carbon (DLC) thin films composed of sp2- and sp3-hybridized carbon show relatively high hardness, excellent biocompatibility, chemical inertness, and high corrosion resistance. By introducing impure elements, such as N and Ni, the doped DLC films can possess sufficient conductivity for electrochemistry, low double-layer capacitance, large potential window, low background current, high stability in challenging environments, and comparative resistance to deactivation by fouling [19]. Experimental results suggest that modified diamond-like carbon electrodes with a wide electrochemical window and low background current can be used as non-enzymatic glucose detection sensors [19,20,21]. In the meantime, DLC films can reduce internal stress to improve the quality of the films, and their performance can be efficiently improved and enhanced. More importantly, DLC can be prepared at low temperatures, has a very wide selectivity to the substrate, and does not require special treatment of the substrate, making it possible to further modify the TiO_2_ nanotubes without damaging the original TiO_2_ nanotube structure. In addition, metallic nanoparticles (NPs) or nanoclusters (NCs) loaded on the electrode surface represent obvious differences in physical and chemical properties from both substrate materials and isolated bulk metals and drastically increase the catalytic activity and sensitivity of the electrodes, resulting in the enhancement of the performance of biosensors [22,23,24]. Large surface areas and good electronic properties of dispersive metallic particles, such as Au and Ni, can accumulate charges and improve the electrochemical response of carbon electrodes in electrochemical glucose sensors [25,26,27,28].

In this work, we use the sputtered DLC layer to act as a fixed tool that is supposed to trap the Ni nanoparticles. The metal particles could be evenly dispersed and fixed within the carbon film, and the smooth surface can reduce the adsorption oxidation products. Sputtering at a substrate could form a local chemical combination of a two-phase interface, reducing the electron transfer resistance and improving the catalytic activity [29].

Therefore, we prepared TiO_2_ nanotubes (TNTs) using the anodic oxidation process and then modified the Ni-doped DLC film layer on the surface of the TiO_2_ electrode using magnetron sputtering technology to prepare the Ni-doped DLC-modified TiO_2_ nanotube composite structure electrodes (Ni-DLC/TNTs). Their structural composition and electrocatalytic oxidation performance of glucose were systematically investigated.

## 2. Results and Discussion

### 2.1. Electrode Characteristics

**Figure 1:**
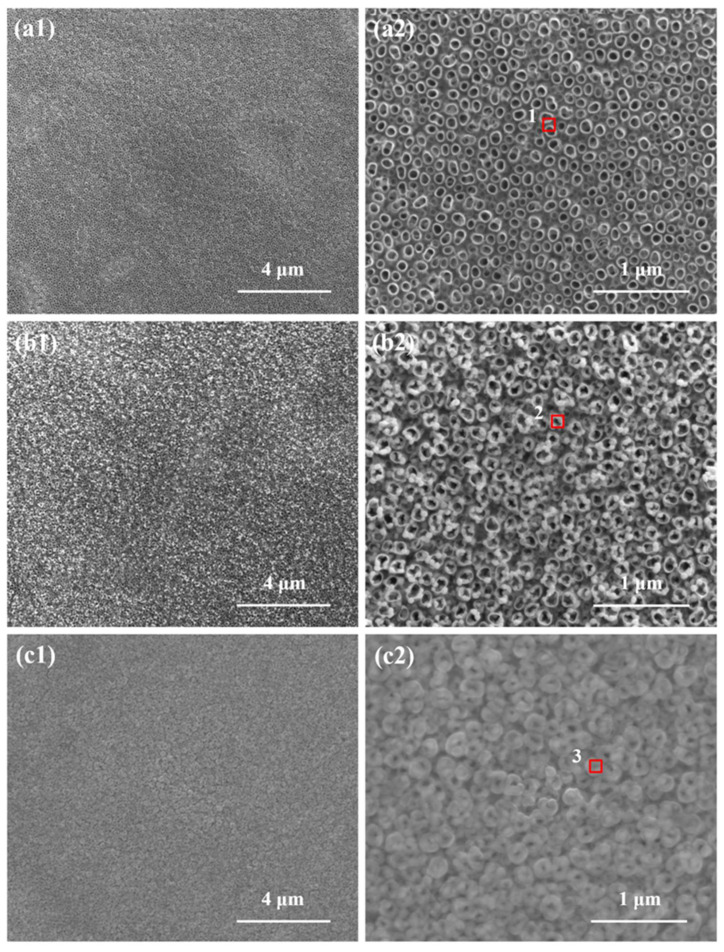
SEM morphology of different electrode surfaces: (**a**1,**a**2) TNT; (**b**1,**b**2) Ni/TNT; and (**c**1,**c**2) Ni−DLC/TNT membrane layers. The elemental analysis in Figure 2 demonstrates the successful deposition of Ni with an atomic ratio of 4.1% for Ni/TNTs and 6.13% for Ni−DLC/TNTs, respectively, after magnetron sputtering. The three red small box identifier showed that after the deposition of Ni nanoparticles, the Ni NPs were dispersed and uniformly distributed on the surface of the TiO_2_ nanotubes. As can be seen in Figure 1c, after the sputtering deposition of Ni−doped DLC film on the surface of the nanotubes, the opening end of the nanotubes decreased and the sur-face retained the morphology and roughness of the substrate.

Figure 1 shows the SEM morphology of the TNTs, Ni/TNTs, and Ni-DLC/TNTs. As can be seen in Figure 1a, the TiO_2_ nanotubes have an opening at the top and are arranged vertically on the Ti surface in an orderly manner, with a diameter of less than 100 nm. As can be seen in Figure 1b, after the deposition of Ni nanoparticles, the Ni NPs were dispersed and uniformly distributed on the surface of the TiO_2_ nanotubes. This is because the periodic variation in the pulse electroplating current was conducive to eliminating the cathode concentration polarization and greatly improving the nucleation rate. On the other hand, the current was turned on and off during the pulse electrodeposition process. Such periodic alternation is very beneficial to the adsorption and desorption of ions in the electrolyte and the recrystallization of particles [30], which reduces the possibility of nanoparticles, reunion, and fine grains. As can be seen in Figure 1c, after the sputtering deposition of Ni-doped DLC film on the surface of the nanotubes, the opening end of the nanotubes decreased and the surface retained the morphology and roughness of the substrate.

**Figure 2:**
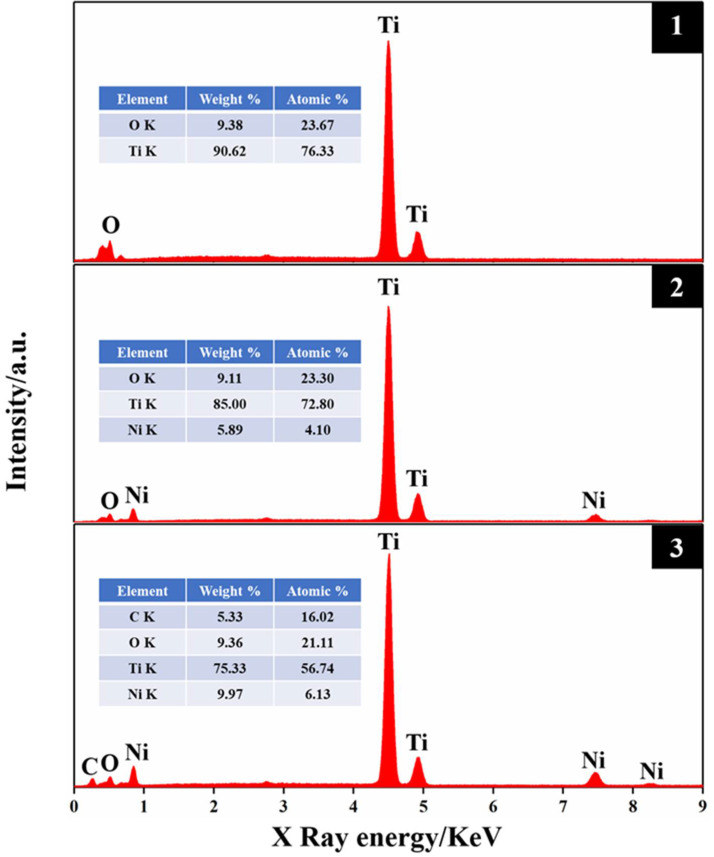
EDX energy spectra 1, 2, and 3 correspond to the regions (red rectangle) marked on the surfaces of the TNT (a2), Ni−TNT (b2), and Ni−DLC/TNT (c2) electrodes in Figure 1, respectively. The illustration shows the types and contents of elements.

The element composition of the electrode was analyzed by EDX spectroscopy. Figure 2 shows the EDX results of the tube on the surfaces of TNTs, TNT-Ni, and TNT-Ni/DLC. EDX can collect the element information at a depth of 1 μm, which is collected in the area of the tube openings, respectively (as marked in Figure 1). Only Ti and O were detected on the surface of the samples without electrodeposition and Ni was detected after electrodeposition. As can be seen in Figure 2, element C was detected on the surface of the TNT-Ni/DLC composite electrode, and it was found that the content of element Ni was greatly increased because the deposited DLC film was doped with Ni at the same time, which improves the performance of the electrode compared to the TNT-Ni electrode.

**Figure 3:**
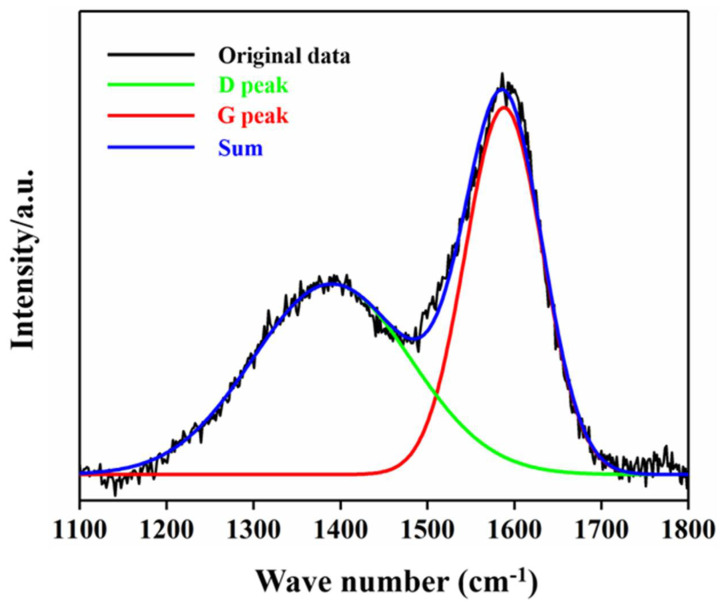
Raman pattern of Ni−DLC/TNT electrode surface.

Figure 3 shows the Raman spectrum of the Ni-DLC/TNT electrode, where a typical diamond-like wide peak with two asymmetric broad peaks can be found. The D peak near the low wavenumber of 1360 cm^−1^ can be derived from the respiration mode of the ring sp^2^ C–C bond, whereas the G peak near the high wavenumber of 1580 cm^−1^ is derived from the stretching vibration mode of the chain-like sp^2^ C–C bond or annular sp^2^ C–C bond [31], indicating the successful deposition of the DLC layer on the electrode surface.

### 2.2. Performance of Electrode Electrocatalytic Oxidation of Glucose

Studies have shown that the catalytic mechanism of a Ni-based sensor is mainly due to the formation of a higher valence hydroxyl compound in alkaline media (NiOOH), which carries out the catalytic oxidation process. The specific reaction is as follows:

Ni + 2OH^−^ → Ni(OH)_2_ + 2e^−^ (1)

Ni(OH)_2_ + OH^−^ → NiO(OH) + H_2_O + e^−^ (2)

NiO(OH) + glucose → Ni(OH)_2_ + glucolactone (3)

**Figure 4:**
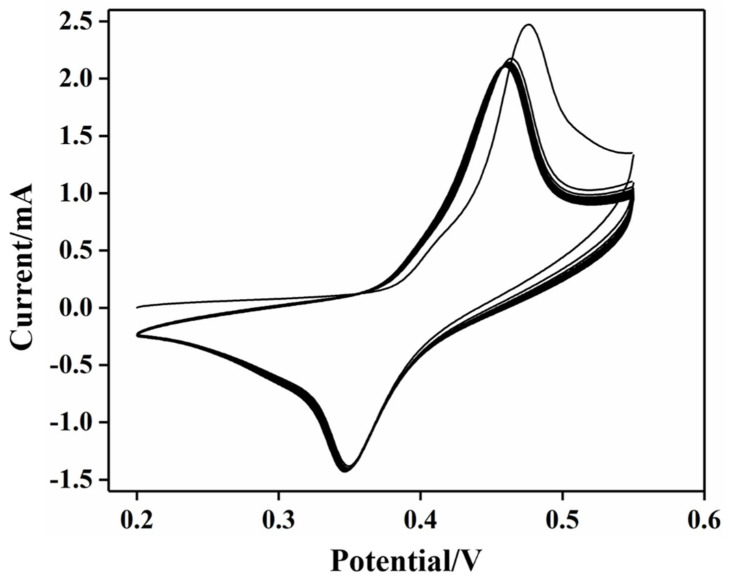
CV curves of Ni−DLC/TNT composite electrode solution were scanned continuously for 25 cycles at a scanning rate of 50 mV s^−1^ (0.5 mol/L sodium hydroxide solution as a medium).

Therefore, before the electrochemical detection of glucose at the electrode, Ni/TNTs and Ni-DLC/TNTs were treated with 25 consecutive cyclic voltammetry (CV) cycles at a scanning rate of 50 mV s^−1^, and Figure 4 shows the CV curve of the Ni-DLC/TNT composite electrode. With the increase in the number of cycles, the oxidation peak current also increased gradually and the oxidation peak potential shifted positively, suggesting the formation of NiOOH.

**Figure 5:**
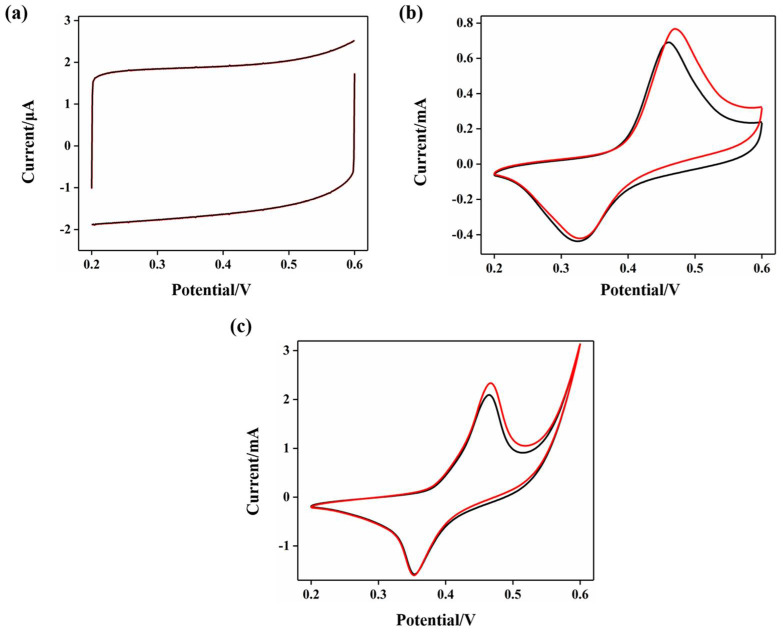
CV curves of different electrodes in 0.5 M NaOH + 1 mM glucose mixed solution (red line) and 0.5 M NaOH solution (black line): (**a**) TNTs; (**b**) Ni−TNTs; (**c**) Ni−DLC/TNTs (all selected the second cycle of CV).

Figure 5 shows the CV curves of the three electrodes, (a) TNTs, (b) Ni/TNTs, and (c) Ni-DLC/TNTs, in background electrolyte 0.5 M NaOH and 1 mM glucose (0.5 M NaOH) solutions, respectively, with a scan rate of 50 mV s^−1^. It can be seen that pure TiO_2_ nanotubes showed no redox activity. After modification with Ni nanoparticles, the Ni/TNT electrode had a Ni^2+^/Ni^3+^ redox peak. However, after modification with Ni-doped DLC film, the current of the redox peak greatly increased. This illustrates that the conductivity of the Ni-DLC/TNT electrode was further improved with the incorporation of DLC. This is because the deposition of the uniformly distributed Ni on the DLC increased the catalytic capacity and the steric resistance of the film, inhibiting the formation of the sp^3^ bond and increasing the proportion of sp^2^ [31,32,33]. When 1 mM glucose was added to the NaOH solution, the TNT electrode showed no response as pure TiO_2_ nanotubes have no catalytic activity for the oxidation of glucose, whereas the peak currents of the Ni/TNT and Ni-DLC/TNT electrodes increased. This is because, in the alkaline solution, the glucose was rapidly and specifically oxidized to gluconolactone in the presence of the Ni catalyst. To be specific, Figure 5b,c shows that the electrocatalytic oxidation response current of the Ni/TNTs was 0.07 mA, which increased to 0.21 mA for the Ni-DLC/TNTs, indicating enhanced activity of the Ni-DLC/TNTs.

**Figure 6:**
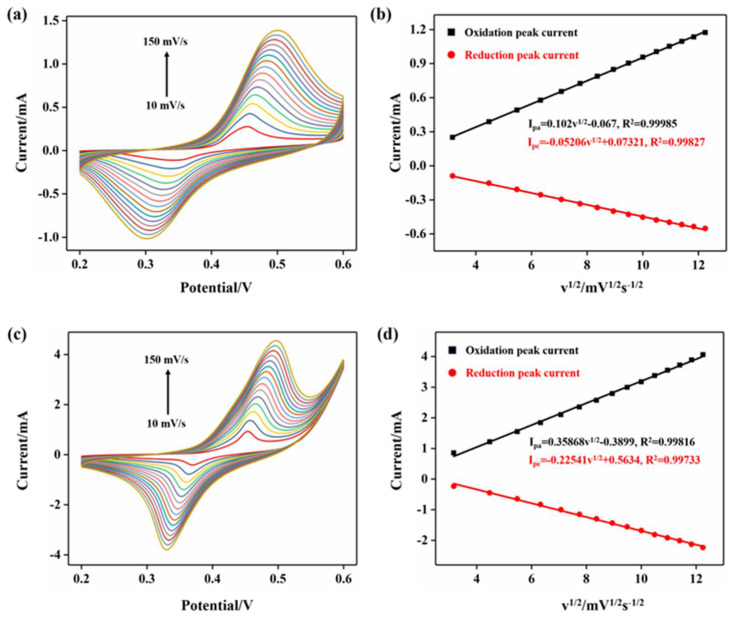
The CV curves of the Ni−TNT electrode (**a**,**b**) and (**c**,**d**) the Ni−DLC/TNT composite electrode at a scanning rate of 10−150 mV/s, the linear fitting graphs of the corresponding anode (I_pa_) and cathode oxidation peak current (I_pc_), and the square root of the scanning rate v^1/2^. TNT electrodes have no catalytic activity.

In order to further explore the mass transfer characteristics in the process of electrochemical glucose oxidation, CV tests were carried out at a scanning rate of 10, 20, 30, 40, 50, 60, 70, 80, 90, 100, 110, 120, 130, 140, and 150 mV/s, respectively, whereas the Ni-TNT electrode and Ni-DLC/TNT composite electrodes were treated with a mixture of 0.5 M NaOH and 1 mM glucose solution (0.5 M NaOH), respectively. As shown in Figure 6, with the increase in the scanning rate, the oxidation peak current and reduction peak current increased gradually with reversibility. Moreover, the oxidation peak potential shifted positively, whereas the reduction peak potential shifted negatively, and the potential difference (△Ep) between the oxidation peak potential and reduction peak potential increased gradually. This is because, at a higher scanning rate, the electrode required a larger overpotential to achieve the same electron transfer rate [34]. The oxidation peak current and reduction peak current in the CV curve were plotted as a function of the square root of the scanning rate, where a good linear relationship was found, as seen in Figure 6b,d, indicating that both the Ni-TNT electrode and Ni-DLC/TNT composite electrode detected glucose as a typical electrochemical process of diffusion mass transfer [35].

**Figure 7:**
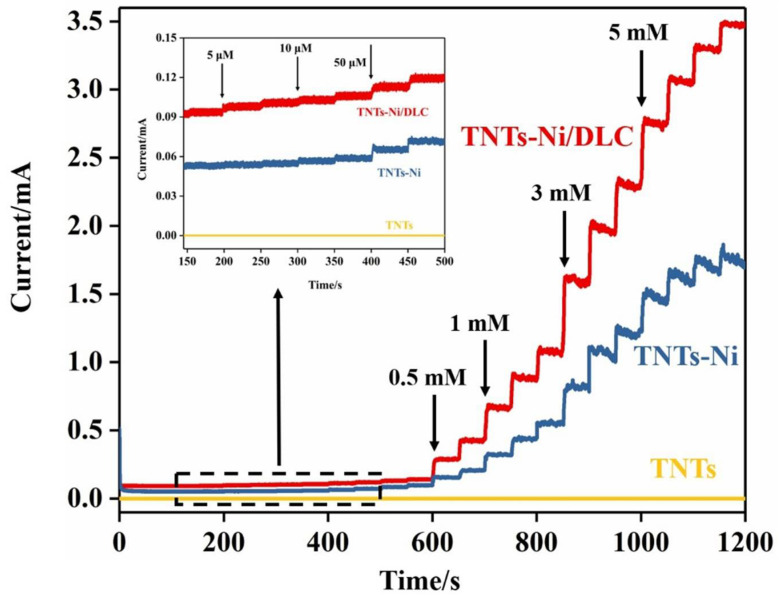
The i-t curves of the TNT electrode, Ni/TNT electrode, and Ni-DLC/TNT composite electrode when different concentrations of glucose solution were continuously added. The illustration shows the enlarged view at low concentrations.

In order to compare the electrocatalytic activity of the TNT electrode, Ni-TNT electrode, and Ni-DLC/TNT composite electrode for glucose, chronoamperometry was employed. The obtained i-t curve results are shown in Figure 7 with an applied potential of 0.55 V. The solution was kept in a state of constant agitation during the test. It can be seen from the i-t curve that the current response of the TNT electrode is a straight line, which indicates that pure TiO_2_ nanotubes do not have the ability to catalyze the oxidation of glucose, whereas the Ni-TNT and Ni-DLC/TNT composite electrodes have a relatively larger response current. The current response of the catalytic oxidation of glucose at each electrode is as follows: Ni-DLC/TNTs > Ni-TNTs > TNTs, which is consistent with the CV results in Figure 5.

**Figure 8:**
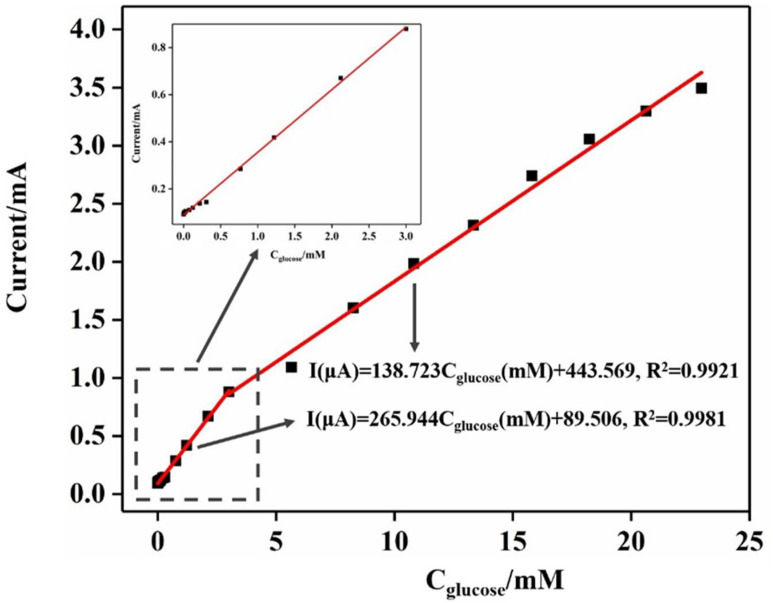
The linear fitting curve and corresponding fitting equation of the current response of the Ni-DLC/TNT composite electrode and the corresponding glucose concentration are shown in the illustration as the enlarged fitting diagram at a low concentration.

The performance of the Ni-DLC/TNT composite electrode for the electrocatalytic oxidation of glucose was further analyzed as shown in the i-t curve. It can be seen in Figure 8 that the current response can reach a stable state within 5 s, indicating that the electrocatalytic oxidation of glucose by the Ni-DLC/TNT composite electrode is rapid. With the increase in the concentration of glucose in the solution, the current response of the electrode also increased step by step. As can be seen from the illustration in Figure 7, when the glucose concentration was lower than 5 μM, the electrode still generated an obvious current response, indicating an excellent sensitivity of the Ni-DLC/TNT composite electrode at a low concentration of glucose.

A comparison of the obtained electrodes and literature on similar non-enzymatic glucose sensors can be seen in Table 1. Based on this comparison, the composite electrode has better selectivity and stability than other similar electrodes in the literature.

Taking the concentration of glucose in solution as the abscissa and the corresponding current response as the ordinate, Figure 8 was plotted. Linear fitting was performed to obtain two linear fitting curves in the ranges of high and low concentrations:

(1) When the concentration of glucose ranged from 0.99 mM to 3.00 mM, the linear fitting equation was as follows: I (μA) = 265.944 Cglucose(mM) + 89.506, and the linear correlation coefficient was R2 = 0.9981.

(2) When the concentration of glucose ranged from 3.00 mm to 22.97 mM, the linear fitting equation was as follows: I (μA) = 138.723 Cglucose(mM) + 443.569, and the linear correlation coefficient was R2 =0.9921.

Since the sensitivity is the ratio of the slope of the linear fitting curve to the electrode area and the physical surface area of the electrode was 0.25 cm^2^, the sensitivity of the electrode was estimated to be 1063.78 μA·mM^−1^·cm^−2^ with a glucose concentration range between 0.99 mM and 3.00 mM, whereas the sensitivity of the electrode was 554.89 μA·mM^−1^·cm^−2^ and in the range of 3.00–22.97 mM. The reason the sensitivity of the electrode decreased in a high concentration range can be attributed to the following reasons: The intermediate products generated by the oxidation of glucose were adsorbed on the surface of the composite electrode during the electrochemical testing process, leading to the obstruction and inhibition of the adsorption and diffusion of glucose molecules to the surface of the electrode [46]. In addition, according to the background current signal measured by the electrode in a blank solution 11 times, the standard deviation was 0.18 μA·cm^−2^. According to the calculation formula of the detection limit, LOD = 3σ/S, with S the sensitivity and σ the relative standard deviation, and with a principle of S/N = 3, the detection limit of the Ni-DLC/TNT composite electrode was calculated to be 0.53 mM.

**Figure 9:**
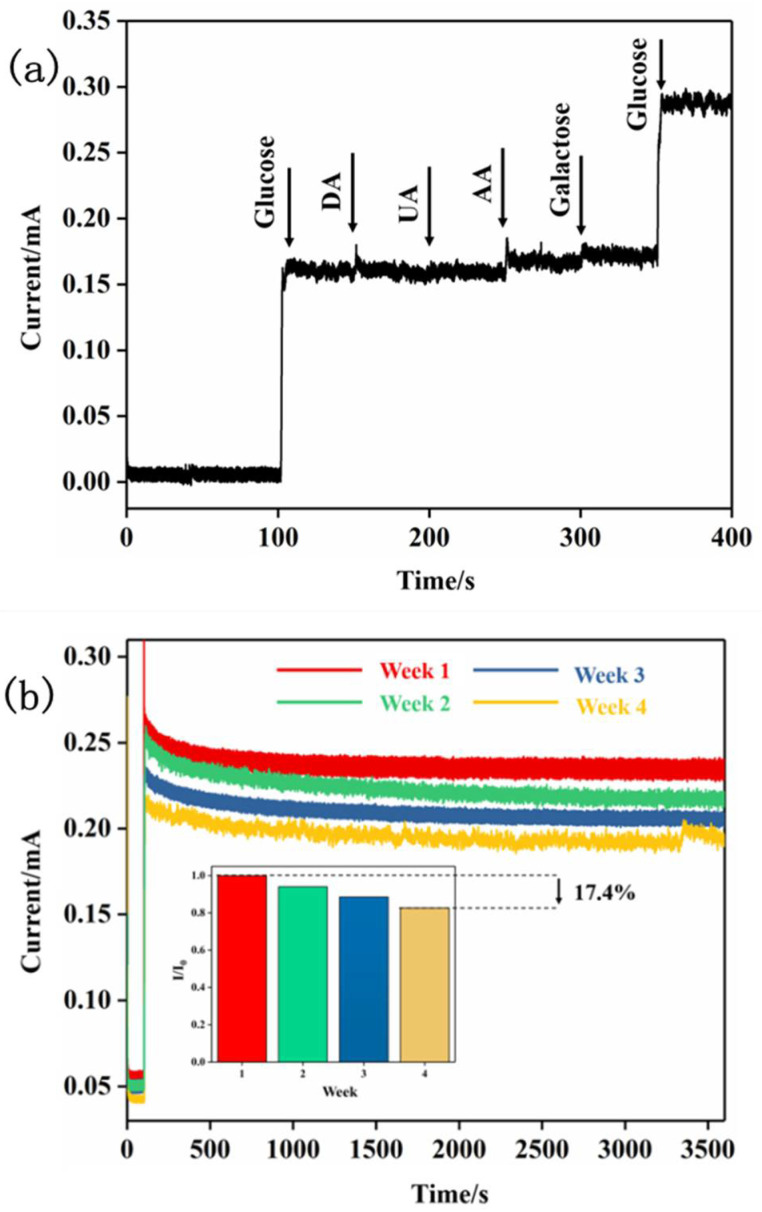
(**a**) Anti-interference test results of Ni-DLC/TNT composite electrode; (**b**) Stability test of TNT-Ni/DLC composite electrode. The i-t curve in 1 mM glucose solution was measured once a week. The illustration shows the results of each test relative to the first test.

Another major problem faced by non-enzymatic sensors is the interference of other organic substances in the blood because these substances can be oxidized with a potential similar to glucose; therefore, we further investigated the anti-interference performance of the TNT-Ni/DLC composite electrode. In solution, the chronoamperometry method was used and the applied potential was 0.55 V, glucose (1 mM), DA (0.1 mM), UA (0.1 mM), AA (0.1 mM), galactose (0.1 mM), and glucose (1 mM) were added in turn to study their influence on the current response and the results are shown in Figure 9a.

It can be seen from the results that other than glucose, the interferential current signals compared to the current signal of glucose were almost negligible, suggesting a strong anti-interference of the electrode. Under a 0.55 V voltage, the electrodes only showed weak oxidation ability to other interfering substances in the blood of the human body, which ensured accuracy in the process of biological detection.

The long-term stability of the electrode is also an important index to determine whether the electrode has application potential. The current response of the Ni-DLC/TNT composite electrode in a 1 mM glucose solution was tested by the time current method once a week for four consecutive weeks. During the test, the solution was kept in a state of constant agitation in the supporting electrolyte and the applied potential was 0.55 V. Moreover, the electrode was stored at room temperature in air during the non-test period, and the stability of the electrode was checked over time from the SEM image of the electrode after experiments. The test results are shown in Figure 9b. It can be seen that in each continuous 1 h test process, the current response showed hardly any reduction, and the current response of the composite electrode remained at 82.6% of the initial current response after one month, indicating that the Ni-DLC/TNT composite electrode had good long-term stability. Compared with the electrodeposited nanoparticles and the matrix by adsorption or another physical combination, the Ni in the Ni-DLC/TNT electrode prepared in this paper was closely bound to the DLC, which was firmly fixed in the film by the DLC and formed a uniform distribution. In addition, the hollow TiO_2_ carrier electrode played the role of stable fixation for the DLC film. This avoids the shedding and loss of electrodeposited nanoparticles and the reduction in the current response of the electrode in the electrochemical test, which greatly improves the long-term stability of the electrode.

## 3. Materials and Methods

### 3.1. Reagents

Potassium chloride (0.5 mol/L, 99.97%) and sodium hydroxide (0.5 mol/L, 99.5%) were all purchased from Tianjin Recovery Technology Development Co., Ltd., Tianjin, China, glucose (0.99–3 mM, 99.5%), dopamine (0.1 mM, ≥95%), ascorbic acid (0.1 mM, 99%), uric acid (0.1 mM, 98%), and lactose (0.1 mM, 99.6%) were all purchased from Sigma-Aldrich Co., Ltd., St. Louis, MO, USA. All reagents were analytically pure without any further purification. All solutions were prepared using ultra-pure water (18.2 MΩ ∗ cm). The supporting electrolyte was a 0.5 M NaOH solution.

### 3.2. Sample Preparation

**Figure 10:**
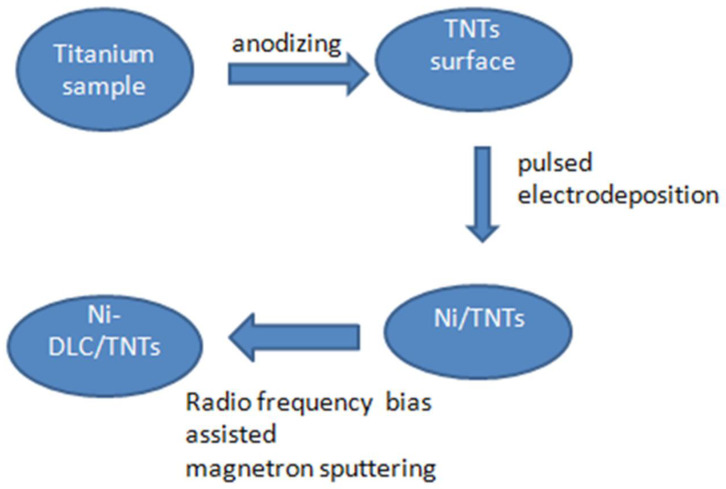
Steps in the sample preparation.

Firstly, the polished Ti sheet (PT 99.99%, Hunan Xiangtian Titanium Industry Co., Ltd., Changsha, China) (5 × 5 × 1.5 mm) was soaked in the chemical polishing solution NaF:HNO_3_:H_2_O = 2:18:80(m/m) for 1–3 min to remove the oxide layer. Then, the treated Ti sheet was cleaned by sonication in deionized water for 5 min and dried. The treated Ti sheet was used as the anode. The TiO_2_ nanotube array (TNTs) electrodes were obtained by anodizing at 25 V for 1 h in glycerol solution containing 0.2–7 M NH_4_F using stainless steel plates as the cathode placed 30 mm away from the anode. Secondly, Ni nanoparticles were deposited on TiO_2_ nanotube arrays using the pulsed electrodeposition method and were prepared according to the literature [47]. The experiment was carried out in a three-electrode system, with the TNT electrode as the working electrode, nickel sheet as the counter electrode, and Ag/AgCl/saturated KCl electrode as the reference electrode. The electrolyte contained 300 g/L NiSO_4_·6H_2_O, 45 g/L NiCl_2_·6H_2_O, and 37 g/L H_3_BO_3_, and the temperature was maintained at 38 °C. The cathode pulse current density and time were −160 mA/cm^2^ and 8 ms, the anode pulse current density and time were set at +160 mA/cm^2^ and 2 ms, the current turn-off time was 1000 ms, and the total deposition time was 10 min. After electrodeposition, the sample was rinsed with deionized water and dried. Finally, Ni-doped DLC films (Ni-DLC/TNTs) were prepared on the surface of the TNT electrode using a radio frequency (RF) bias-assisted magnetron sputtering process. The specific deposition process was as follows: The flow ratio of Ar gas (99.999%) and C_2_H_2_ (99.999%) was 16:6 sccm, the deposition pressure was 1.0 Pa, the RF power was 200 W, the bias voltage was 25 V, and the sputtering time was 5 min. The steps in the sample preparation are shown below in Figure 10.

### 3.3. Sample Characterization and Electrochemical Detection

The surface morphology of the samples was analyzed by field emission scanning electron microscopy (FESEM, NOVA Nanosem230), the surface composition of the samples was characterized by energy-dispersive X-ray spectroscopy (EDS), and X-ray photoelectron spectroscopy (XPS, ESCALAB 250Xi) was used to characterize and analyze the chemical composition of the surface of the samples. Laser Raman spectroscopy (Labram HR 800,532 nm, 10 mW) was used to characterize the proportion of sp^2^ and sp^3^ in the DLC samples. All the electrochemical performance characterization experiments were performed at the electrochemical workstation CHI660E (CH Instruments, Shanghai Chenhua Instrument Corp., Shanghai, China) at room temperature using a three-electrode system. The packaged sample (5 × 5 mm^2^) was used as the working electrode, a platinum plate (10 × 10 mm^2^) was used as the counter electrode, and an Ag/AgCl was used as the reference electrode.

## 4. Conclusions

In this manuscript, TiO_2_ nanotubes were prepared by anodic oxidation, and the composite electrode modified by Ni-DLC was prepared by Ni electrodeposition and subsequent DLC sputtering. With TiO_2_ as the substrate electrode, the hollow porous structure improved the stability of the modifying film by anchoring. At the same time, the synergistic effect of Ni and DLC improved the stability and catalytic activity of Ni. The results of the glucose catalytic oxidation of the composite electrode showed that there were two linear ranges. The sensitivity of the composite electrode was 1063.78 μA·mM^−^^1^·cm^−2^ when the glucose concentration range was 0.99 mM–3.00 mm, the sensitivity of the electrode was 554.89 μA·mM^–^^1^·cm^–2^, and the LOD was 0.53 μM (S/N = 3) in a glucose concentration range of 3.00–22.97 mM. The composite electrode had good selectivity and stability, which indicates that the electrode has positive application prospects in the determination of non-enzymatic glucose.

## Figures and Tables

**Table 1:** Comparison of the performance of the proposed sensor and those of similar ones described in the literature.

Electrode Material	Linear Range	Detection Limit (μM)	Sensitivity (μA·mM^−1^·cm^−2^)	Ref.
Ni-NDa/BDD	0.2–12 μM; 31.3 μM–1.06 mM	0.05	120; 35.6	[36]
NiNPs/GNc	5 μM–0.55 mM	1.85	865	[12]
NiONPs/GOd/GCE	3.13 μM–3.05 mM	1	1087	[37]
NiO/Pt/ErGO/GCE	50 μM–5.66 mM	0.2	668.2	[38]
Ni-MWCNTe/GCE	3.2 μM–17.5 mM	0.89	67.19	[39]
Ni(OH)2graphene/GCE	1–10 μM; 10 μM–10 mM	0.6	494; 328	[40]
NiCoO/CNTf	0.01–0.93 mM; 0.93–12.12 mM	5	66.15; 15.43	[41]
NiNPs/BDD	10 μM–10 mM	2.7	1040	[42]
NiNPs/CNFg	2 μM–2.5 mM	1	420.4	[7]
Ni-Rgo/GCE	1–110 μM	-	813	[18]
Au/DLC:P	0.5–25 mM	300	37	[19]
Au-DLC:N	0.5–25 mM	120	-	[20]
CuO	0.005–7.95 mM	1	622.2	[22]
AuNi/NX/MWCNT-21	1–60 mM	0.063	662.93	[43]
	60–1900 mM	0.285	147.22	
Ni-NPs/GRE	2–800 mM	0.4	1387	[44]
Cu-Ni/NF	1−600 mM	2	11,340.25	[45]
Ni-DLC/TiO_2_	0.99–22.97 mM	0.53	1063.78	This work

## Data Availability

Not applicable.

## Sample Availability

Samples of the compounds are not available from the authors.
